# The multicausal twilight of South American native mammalian predators (Metatheria, Sparassodonta)

**DOI:** 10.1038/s41598-022-05266-z

**Published:** 2022-01-24

**Authors:** Sergio Daniel Tarquini, Sandrine Ladevèze, Francisco Juan Prevosti

**Affiliations:** 1 Centro Regional de Investigaciones Científicas y Transferencia Tecnológica de La Rioja (CRILAR - Provincia de La Rioja, UNLaR, SEGEMAR, UNCa, CONICET), Entre Ríos y Mendoza s/n, F5301 Anillaco, La Rioja Argentina; 2 Centre de Recherche en Paléontologie - Paris (CR2P - CNRS, MNHN, Sorbonne Université), 8 rue Buffon, 75005 Paris, France; 3 Museo de Ciencias Antropológicas y Naturales - Universidad Nacional de La Rioja (UNLaR), Av. Luis M. de la Fuente s/n, Ciudad Universitaria de la Ciencia y de la Técnica, F5300 La Rioja, Argentina; 4 Departamento de Ciencias Exactas, Físicas y Naturales, Universidad Nacional de la Rioja (UNLaR), Av. Luis M. de la Fuente s/n, Ciudad Universitaria de la Ciencia y de la Técnica, F5300 La Rioja, Argentina; 5 Consejo Nacional de Investigaciones Científicas y Técnicas (CONICET), Ciudad Autónoma de Buenos Aires, Argentina

**Keywords:** Palaeontology, Biodiversity

## Abstract

Sparassodonts were the apex mammalian predators of South America throughout most of the Cenozoic, diversifying into a wide array of niches including fox-like and even saber-toothed forms. Their extinction is still controversial, with different authors suggesting competition with other predators (placental carnivorans, terror birds, and carnivorous opossums), extinction of prey, and climate change as causal explanations. Here, we analyse these hypotheses using a novel approach implicating Bayesian analyses. We find that speciation and extinction rates of sparassodonts can be correlated with (i) intrinsic biotic factors such as changes in body mass and diversity of sparassodonts, (ii) extrinsic biotic factors such as potential prey diversity, and iii) extrinsic abiotic factors like the atmospheric CO_2_, sea level, temperature, and uplift of the Andes. Thus, sparassodonts are a good example of a multilevel mixed model of evolution, where various factors drove the evolutionary history of this clade in a pluralistic way. There is no evidence for competition between Sparassodonta and others predators, and the effect of competition in the face of extinctions of fossil species should be tested and not assumed. Furthermore, we propose a novel approach for evaluating the fossil record when performing macroevolutionary analyses.

## Introduction

From the beginning of the Cenozoic until present times, the fauna of South America has undergone considerable changes throughout all trophic levels^1–4^. Following the extinction of the nonavian theropod dinosaurs, the predominant terrestrial apex predators in South America were giant snakes (Boidae, Madtsoiidae), terrestrial crocodyliforms (Sebecidae), terror birds (Phorusrhacidae), and metatherian mammals (Sparassodonta)^3,5–11^. These groups were the dominant terrestrial carnivores in South America until about 3 Ma, at which point their niches subsequently became occupied by placental mammals (Carnivora)^5–9^. Sparassodonta are a group of endemic metatherians (the clade including marsupials and all the taxa more closely related to marsupials than to placentals) that inhabited South America presumably between 65 and 3.04 Ma^12–14^. This clade included carnivorous and insectivorous mammals, which ranged in size from 250 g to more than 200 kg, and played different roles in the ecosystem of the Cenozoic^5,8^.

Faunal changes in South American mammal communities have accompanied the geological and environmental changes that occurred on the continent during the past 65 Ma. At the beginning of the Cenozoic, South America was still connected to Antarctica and indirectly to Australia^15,16^ and as a result organisms could still disperse between these continents, as evidenced by the shared presence of several groups such as native ungulate mammals (astrapotheres and litopterns) and small metatherians (microbiotheres and polydolopids) on both landmasses^17–19^. Another geological event in this Era was the Andean uplift. The uplift of the Andes has a complex geological history, with regions of this mountain range being uplifted at different latitudes at different times^20,21^. Between 30 and 20 Ma, a first pulse of elevation likely generated very high mountain ranges (more than 50% of their current height) in the Central Andes, which created geographical barriers to winds and moisture^22,23^. Finally, in more recent times (< 3 Ma), the formation of the Isthmus of Panama allowed for a land connection between South and North Americas and a new dispersal of biota between continents^24–26^. These tectonic processes changed climate at a global and local level because of a rearrangement of the oceanic currents and changes in the pattern of rain and winds^15,22,27,28^.

Understanding the changes in biodiversity through time is crucial to identify which mechanisms are involved in the decline or thrive of clades. Three macroevolutionary models have been proposed to explain diversity dynamics: (1) the Red Queen model, in which changes in speciation and extinction rates are due to biotic factors, either intrinsic factors within a species (*e.g.*, body mass) or the interspecific interactions between species (*e.g.*, competition); (2) the Court Jester model, where the changes are dominated by abiotic or extrinsic factors (*e.g.*, climate change); and (3) the Multilevel Mixed model which is a combination of both^29,30^. Traditionally, the extinction of clades was explained as due to solely biotic or abiotic factors, but it is now understood that these factors more likely operate jointly and have differing influence depending on the scale of the analysis^29,31^. The extinction of sparassodonts has long been considered as resulting from ecological competition, whether it be competition with immigrant placental carnivorans during the Great American Biotic Interchange^1,32,33^, or competition with autochthonic predator groups such as carnivorous opossum (namely Didelphini, Sparassocynini, and *Thylatheridium*)^5,34–36^, or the terrestrial avian family Phorusrhacidae^5,6^. However, the decline of sparassodonts has also been suggested to be been driven by other biotic factors, such as extinction of their prey base^5,7,37^, or by abiotic factors, such as climate change^5,7,38,39^. Recent studies reanalysed the competition hypotheses using paleontological inferences of diversity and ecological overlap, and concluded that these hypotheses were unsupported^7,8,37,39–44^. However, few quantitative analyses have tested the other hypotheses. Some studies detected a positive relationship between the diversity of sparassodonts and global temperature^8,39^, but no correlation between prey body mass and diversity of sparassodonts^8^. Furthermore, López-Aguirre et al.^37^ correlated the diversity of Sparassodonta with different variables and concluded, by means of multiple regression analysis, that the diversity of three specific mammal groups correlated with diversity of sparassodonts: Didelphimorphia (*i.e.*, opossums and relatives), African migrants (*i.e.*, caviomorph rodents and platyrrhine primates), and South American ungulates (*i.e.*, astrapotheres, litopterns, notoungulates, pyrotheres, and xenungulates). However, all these analyses were based on the numerical count of sparassodont species rather than evolutionary rates. In recent years Silvestro et al.^45^ have proposed new Bayesian methods to directly calculate speciation and extinction rates from the fossil record, considering biases in the preservation. Furthermore, these rates can be correlated with different variables to test biological interaction hypotheses like competition (*e.g.*, Red Queen model) and the effect of environmental changes (*e.g.*, Court Jester model)^46^. This contribution aims to study the evolution of Sparassodonta using these new Bayesian approaches and to test the different proposed hypotheses about the causes of its extinction.

## Results

### Rates of evolution of Sparassodonta using a Bayesian framework

We evaluated four datasets of the sparassodont fossil records (see “Material and Methods” for more details): (1) a dataset of all occurrences evaluated at the species level dated with precise methods (hereafter species occurrence dataset), (2) a dataset of all sparassodont specimens identified at the species level with precise dating (hereafter fossil specimens dataset), (3) a dataset of occurrences at the species level dated with the boundaries of the South American Age/Stage (hereinafter South American Age/Stage dataset), and (4) a dataset of occurrences at the genus level with precise dating (hereafter genera dataset).

**Figure 1:**
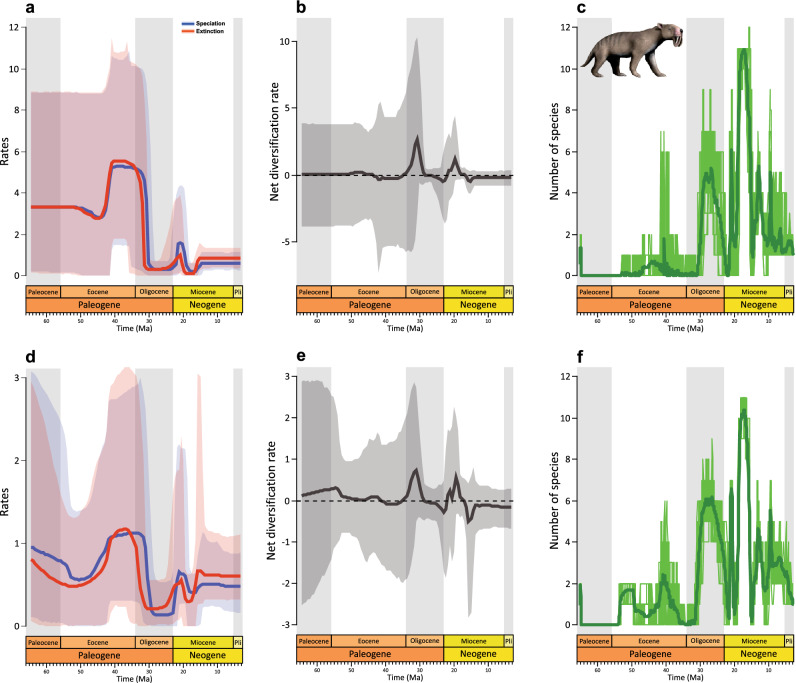
The rise and fall of Sparassodonta throughout the Cenozoic. The Bayesian estimates of speciation (blue), extinction (red), net diversification (grey, speciation minus extinction) rates, and diversity based on species occurrence dataset (**a**–**c**) and fossil specimens dataset (**d**–**f**). Solid lines indicate mean rates, and the shaded areas show 95% credible intervals. *Thylacosmilus atrox* designed by Nobu Tamura (Wikimedia Commons): https://creativecommons.org/licenses/by-sa/4.0/.

Both the species occurrence and fossil specimens datasets show similar changes in speciation and extinction rates through the Cenozoic (Fig. 1a,d). High net diversification rate (*i.e.*, speciation rate minus extinction rate) are observed in the early Oligocene and early Miocene (Fig. 1b,e). Additionally, confidence intervals for speciation, extinction, and net diversification rates are very wide for ages pre-dating the late Oligocene (> 30 Ma), whereas confidence intervals become much narrower for late Oligocene-Pliocene intervals (Fig. 1a,b,d,e). Sparassodont diversity was highest in the late Oligocene and late early Miocene (Fig. 1c,f). The beginning of the demise of Sparassodonta started *circa* 17 Ma when the extinction rate exceeded the speciation rate, resulting in a negative net diversification rate (Fig. 1a,b,d,e). From the middle Miocene onward, the net diversification rate was never positive again, and the speciation and extinction rates remained relatively constant for the rest of their evolutionary history in the species occurrences dataset (Fig. 1a,b,d,e). In the fossil specimens dataset, the net diversification rate decreases even more at 9 Ma, mostly driven by decreases in speciation rate (Fig. 1d–e). In the case of the South American Age/Stage dataset, the curves follow the same pattern, although the peaks and valleys are more pronounced (supplementary information 1, Figs. S1a-c). In the case of the genera dataset the curves flatten from the Eocene, only registering an increase in the extinction rate from 16 Ma (supplementary information 1, Figs. S1d-f).

### Correlations between rates of evolution and body mass

**Figure 2:**
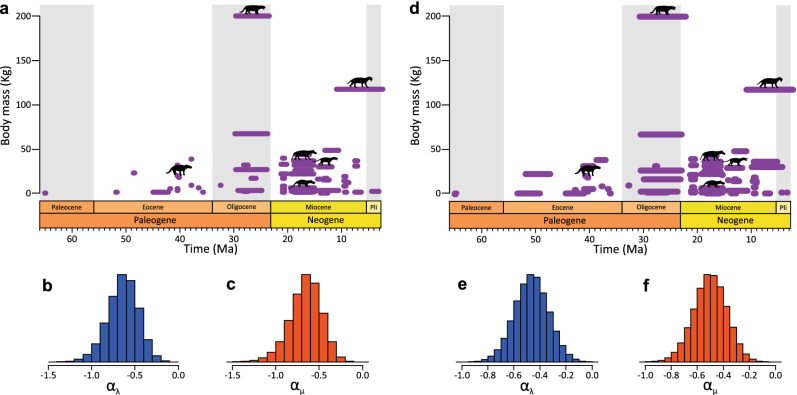
Correlations between diversification dynamics and body mass evolution in Sparassodonta. Analysis from the species occurrence dataset (**a**–**c**) and the fossil specimens dataset (**d**–**f**). The reconstructed life span of species obtained from one of 20 replicated analyses from the species occurrence dataset (**a**) and the fossil specimens dataset (**d**). Correlation between changes in body mass and speciation rate (α_λ_) from the species occurrence dataset (**b**) and the fossil specimens dataset (**e**). Correlation between changes in body mass and extinction rate (α_μ_) from the species occurrence dataset (**c**) and the fossil specimens dataset (**f**). Representative silhouettes of Sparassodonta with different body mass are taken from PhyloPic: *Borhyaena tuberata*, *Callistoe vincei*, *Cladosictis patagonica*, *Lycopsis longirostrus*, *Proborhyaena gigantea*, and *Thylacosmilus atrox* designed by Zimices and licensed under the Creative Commons Attribution 3.0 Unported license (http://creativecommons.org/licenses/by/3.0/).

Small-bodied sparassodonts were present throughout the entire evolutionary history of the clade: from the small early Paleocene mayulestids (*Allqokirus australis* and *Mayulestes ferox*; body masses around 0.3 kg) to the last hathliacynids (*Borhyaenidium riggsi* and *Notocynus hermosicus*; body masses about 2–2.5 kg) in the Pliocene (Fig. 2a,d). By contrast, large sparassodonts first appear in the middle Eocene^47^, with animals even exceeding 30 kg (such as *Arminiheringia auceta*). But it was not until the Oligocene that the largest sparassodonts appeared, such as *Proborhyaena gigantea* (200 kg) and *Australohyaena antiqua* (about 70 kg). The last large sparassodont was the saber-toothed marsupial *Thylacosmilus atrox* (about 120 kg), which is recorded in the Late Miocene-Pliocene (Fig. 2a,d). Body mass is found to be negatively correlated to both speciation (${\overline{\text{x}}}_{{\upalpha \uplambda }}$ = − 0.63; Fig. 2b) and extinction rates (${\overline{\text{x}}}_{{\upalpha \upmu }}$ = − 0.65; Fig. 2c), based on the species occurrence dataset. The same pattern is observed for the other dataset (${\overline{\text{x}}}_{{\upalpha \uplambda }}$ = − 0.47 and ${\overline{\text{x}}}_{{\upalpha \upmu }}$ = − 0.50; Fig. 2e–f). That is*,* as body size decreases, speciation and extinction rates both increase (Fig. 2).

### Correlations between rates of evolution and external variables

**Figure 3:**
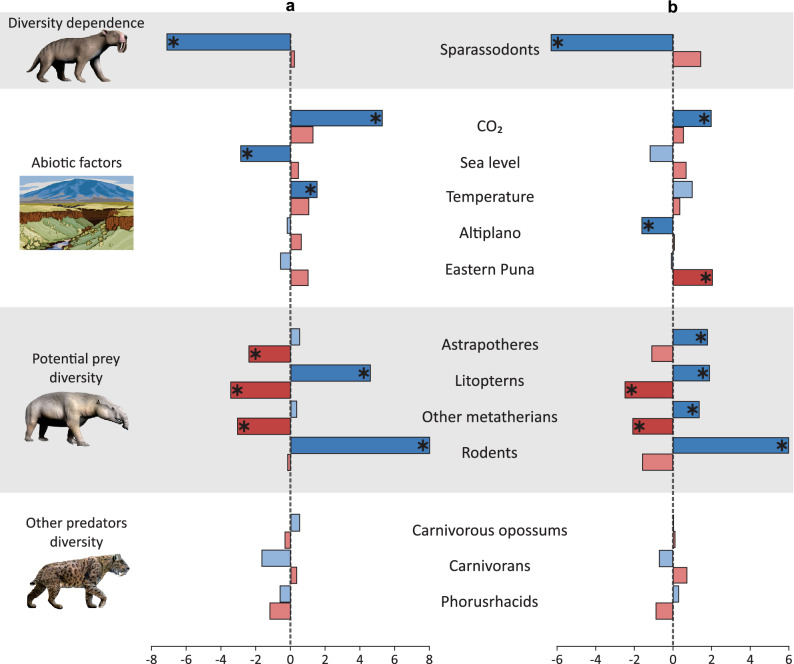
Paleoenvironmental correlations. Correlation parameters for Sparassodonta speciation (G_i_, in blue) and for Sparassodonta extinction (G_m_, in red) with the species occurrence dataset (**a**) and the fossil specimens dataset (**b**). Significant correlations (w > 0.5) are indicated by asterisks and darker colours. Landscape illustrated by Gordon Johnson (Pixabay). *Thylacosmilus atrox* and *Astrapotherium magnum* designed by Nobu Tamura (Wikimedia Commons) and *Smilodon fatalis* designed by Dantheman9758 (Wikimedia Commons): https://creativecommons.org/licenses/by-sa/4.0/.

We used a Multivariate Birth–Death model (MBD)^46^ to evaluate the correlation of different external variables with speciation and extinction rates of both the species occurrence dataset and the fossil specimens dataset (see “Material and Methods” for more details). Based on the species occurrence dataset, the analysis found the following significant correlations, in which shrinkage weights (w*)* is greater than 0.5 (all the correlation parameters being available in supplementary information 1, Table S1). The linear correlation model indicates the speciation rate of Sparassodonta is positively correlated with the diversity of Litopterna (G_i_ = 4.60, w = 0.65), diversity of Rodentia (G_i_ = 8.01, w = 0.54), global atmospheric CO_2_ (G_i_ = 5.28, w = 0.64), and global temperature (G_i_ = 1.53, w = 0.51) (Fig. 3a). Inversely, the speciation rate of Sparassodonta was negatively correlated with both diversity of Sparassodonta (G_i_ = − 7.12, w = 0.88) and global sea level (G_i_ = − 2.87, w = 0.57) (Fig. 3a). The extinction rate of Sparassodonta was negatively correlated with the diversity of various potential prey: Astrapotheria (G_m_ = − 2.40, w = 0.55), Litopterna (G_m_ = − 3.45, w = 0.61), and other Metatheria (G_m_ = − 3.05, w = 0.59) (Fig. 3a). There is no significant correlation (all w less than 0.5) between the diversity of other predators and the rates of speciation or extinction of Sparassodonta (Fig. 3a).

Based on the fossil specimens dataset (all the correlation parameters are in supplementary information 1, Table S2), the respective linear correlation model indicates positive correlations between the speciation rate of Sparassodonta and both global atmospheric CO_2_ (G_i_ = 1.98, w = 0.52) and diversity of various potential prey: Astrapotheria (G_i_ = 1.79, w = 0.52), Litopterna (G_i_ = 1.89, w = 0.55), other Metatheria (G_i_ = 1.35, w = 0.50), and Rodentia (G_i_ = 5.98, w = 0.54) (Fig. 3b). Inversely, the speciation rate of Sparassodonta was negatively correlated with both diversity of Sparassodonta (G_i_ = − 6.30, w = 0.90) and Altiplano paleoelevation (G_i_ = − 1.61, w = 0.50) (Fig. 3b). The extinction rate of Sparassodonta was positively correlated with Eastern Puna paleoelevation (G_m_ = 2.04, w = 0.57), but negatively correlated with both diversity of Litopterna (G_m_ = − 2.48, w = 0.55) and diversity of other Metatheria (G_m_ = − 2.08, w = 0.54) (Fig. 3b). There is no significant correlation (all w less than 0.5) between the diversity of other predators and the rates of speciation or extinction of Sparassodonta (Fig. 3b).

## Discussion and conclusions

The fossil record is by nature incomplete because of various sampling biases: taphonomic biases in how fossils are preserved, geological biases in how those rock layers become exposed, and then anthropogenic sampling biases in when and where fossils are collected^48–54^. The South American fossil record is not unique in this regard, there are marked hiatuses in fossil record mainly during Paleogene, and although there are some exceptions, fossil sites are limited to high latitudes^6–8,55,56^. In the present study, these biases were best observed in the Paleogene^8^, in which sparassodont occurrences are more sparse, resulting in a much larger confidence interval in the inferred evolutionary rates (Fig. 1).

However, in the present study in order to overcome these biases in the fossil record we used Bayesian methods, which is up to now the most reliable methodology for inferring evolutionary rates from the fossil record^57^. According to the mean preservation rates and the estimated heterogeneity parameters obtained, we rely on the inferred rates (see “Material and Methods” for more details). Moreover, we generated four alternative datasets for sparassodonts that contemplate different ways of analysing the fossil record. In previous studies using this methodology, the fossil record was incorporated as species occurrences^46,58,59^, so that the differences between localities (*i.e.*, differences in the number of fossils found, due to differences in the areas exposed and areas sampled^6^) can be balanced. However, in a given locality with fewer sampling, we would consider all the fossil specimens as a single occurrence, even though they could come from different stratigraphic levels. In other words, the use of occurrences is linked to the definition of stratigraphic levels and fossil localities, which change according to the authors who have studied that area. While some authors include several localities within a bounded area, others consider them all part of the same locality. Therefore, it is relevant to incorporate the information provided by the entire fossil record to avoid or alleviate these biases. Finally, the South American Age/Stage allows us to avoid bias for those species present in a single location. It is logical to think that the species have a biochron greater than the punctual dating of the fossil locality. Then, problems due to a low occurrence could be avoided if the South American Age/Stage range is used to model the temporal distribution of each species. In conclusion, the congruence found between the rates of the different datasets supports our results, and we recommend modelling the fossil record in different ways when using these analyses in future works.

The speciation rate of sparassodonts exhibits a negative diversity dependence (*i.e.*, correlates negatively with species diversity of Sparassodonta, Fig. 3), which has also been reported in studies of other organisms^46,60^ and can be explained by the niche differentiation hypothesis. As diversity increases interspecific competition increases and empty niches within an ecosystem become increasingly scarce, and consequently the rate of speciation decreases^61^. However, as highlighted by Moen and Morlon^61^, the correlation between species diversity and decreasing speciation rates can be explained also by geographic factors: after vicariance events, as diversification proceeds and species ranges are subdivided within a geographically limited area, the rate of speciation declines since vicariant speciation loses effect in species with small range sizes. More detailed studies are therefore needed to find out which hypothesis would better explain the diversity dynamics of Sparassodonta.

Changes in sparassodont body mass are negatively correlated with both speciation and extinction rates, with the smaller taxa having shorter durations or species longevity (Fig. 2). A previous study^44^ suggested that small sparassodonts were less frequent in the fossil record, and such a bias could cause shorter durations. However, we analysed the frequency of the fossils along the whole continuum of body masses, and we did not find a clear presence of this bias. Our results could be understood as an expression of molecular changes: smaller sized mammals have higher molecular rates of evolution because they have more generations per unit time^62–65^. Higher molecular evolution rates could ultimately lead to higher speciation rates^66,67^ and higher rates of pseudoextinction, which could be observed as higher extinction rates among fossil taxa. Complementary previous investigations show different results on mammal size-biased selectivity, with three possible scenarios: no size bias^68,69^, greater survivorship in large mammals versus small^70,71^, and greater survivorship in small mammals versus large^72–74^. Our results highlight that large sparassodonts had higher species longevity than coeval small sparassodonts (Fig. 2). This could be an example of Stanley's rule, in which certain species are characterized with high speciation and extinction rates, while others display low speciation and extinction rates^75–77^.

Previous authors have reported a correlation between body mass and hypercarnivory in Sparassodonta^7,13,40,78–80^, with larger species showing shorter and more robust mandibles and lower molars with extremely reduced talonids. Our results indicate that the largest sparassodonts, and then the most carnivorous, simultaneously had higher species longevity. An association between large body masses and more carnivorous habits has also been found in placental carnivores, and could be explained by metabolic constraints: species larger than 20 kg are mostly obligated to hunt larger prey^81–83^. Contrary to our results, in canids the evolution of large size was associated with a decline in species longevity^84^ produced by preferential origination of larger species^85^. However, the correlation between origination rate of canids and body mass is not supported by recent studies^59^.

Marshall^5^, in his pioneer study of the evolution of the assemblage of the South American terrestrial carnivorous animals during the Cenozoic, was unable to conclude if the replacement of sparassodonts by carnivorans, carnivorous opossums, and phorusrhacids were active or passive. However, he suggested competitive exclusion in some cases. Our results support the hypothesis that the diversity of Carnivora did not affect the speciation and extinction rates of Sparassodonta, as suggested by previous authors^7,8,37,38,41,43^. This is evidenced by the lack of significant correlations between the diversity of carnivorans and the rate of speciation and extinction of sparassodonts (Fig. 3). Moreover, the extinction rate of Sparassodonta did not increase when the Carnivora invaded South America (*circa* 7 Ma) (Fig. 1). As a matter of fact, the carnivores that entered South America at this time, that is procyonids, were omnivorous and thus occupied a different ecological niche from the mesocarnivorous to hypercarnivorous coeval sparassodonts^5,7,8,38,43^. By contrast, later carnivorans that would have competed with sparassodonts (mustelids, canids, and felids) only entered South America after the extinction of Sparassodonta^5,7,8,24,38,43,55,86,87^, and hence occupied ecological roles that had been left empty by the already extinct sparassodonts.

Our present study rejects any competition between Sparassodonta and other groups of South American native predators. No model found any correlation between the speciation and extinction rates of Sparassodonta and the diversity of carnivorous opossums or terror birds (Fig. 3). These three predators occupied different habitats. Carnivorous opossums exhibit lower body masses than coeval sparassodonts and show molars less specialized for carnivory^7,36,39,41,42^, indicating the two groups not feed on similar prey. Phorusrhacids likely preferred more open habitats than sparassodonts due to their strong cursorial specializations^78,88–90^. Interestingly then, the diversity of other predators had no effect on the speciation and extinction rates of sparassodonts. The arrival of new predators (such as Carnivora in this case) was long considered as a crucial event to explain the extinction of native predators, but studies in current communities have shown that the relationship is not so causal^91,92^. Particularly, the extinction of thylacine and Tasmanian devil from mainland Australia was not only related to competition with the dingo but most likely was caused by human intensification and climate change^93^. Thus, the repeated argument that eutherians always competitively displace metatherians is probably not the most reliable.

Sparassodonts had a wide range of body masses and therefore different species might have exploited different potential prey. The correlation models found a covariation between the evolutionary rate of sparassodonts and the diversity of astrapotheres, litopterns, other metatherians, and rodents (Fig. 3) in accordance with previous studies^37^. The correlation between the rates of sparassodonts and these other taxa could also be a consequence of the same climatic-environmental limitations of both ecological groups (*e.g.*, astrapotheres and sparassodonts were adapted to warm climates, so when the climate turned colder, both taxa became extinct). However, palaeoecological studies suggest that these herbivorous species were potential prey for sparassodonts^44,78,88^. Rodents and smaller metatherians could have represented prey for a wide number of species. Smaller rodents and metatherians like octodontids and palaeothentids may have been preyed upon by small sparassodonts (*Australogale*, *Cladosictis*, *Perathereutes*, *Pseudonotictis*, and *Sipalocyon*), whereas larger rodents such as dinomyids, neoepiblemids, and hydrochoerine caviids may have been preyed upon by larger species (*Arctodictis*, *Borhyaena*, *Lycopsis*, and *Thylacosmilus*)^44,78,88^. Indeed, broken bones and teeth of the dinomyid “*Scleromys*” *columbianus* and other unidentified rodents have actually been recorded as gut contents in *Lycopsis longirostrus*^94^, and coprolites from probably small sparassodonts have been found to contain remains of octodontoid and/or chinchilloid rodents^95^. Astrapotheres and litopterns could have been preyed upon by the largest sparassodonts such as *Arctodictis*, *Borhyaena*, *Prothylacynus*, and *Thylacosmilus*^78,88^. While the adults of the largest herbivores (*e.g.*, *Astrapotherium* or *Theosodon*) may be excluded from potential prey, sparassodonts could have preyed on the juveniles^78^. Our results indicate that the increase of diversity of astrapotheres, litopterns, other metatherians, and rodents favoured the speciation of sparassodonts, while the decrease in prey diversity favoured the extinction of sparassodonts (Fig. 3). A decrease in the diversity of litopterns and notoungulates is observed from the middle Miocene, while astrapotheres are completely extinct after this time^6,96,97^. In the case of rodents (particularly the large-bodied Dinomyidae, Neoepiblemidae, and Hydrochoerinae), although an increase in diversity is observed from the middle Miocene, then from the early Pliocene the opposite trend is observed, adding to the decrease in diversity of litopterns and notoungulates^6,97–99^. Therefore, these changes, along with others, could have caused or contributed to the extinction of sparassodonts.

Here, the MBD analysis shows a significant correlation between evolutionary rates of Sparassodonta and both environmental factors (CO_2_, temperature, and sea level) and uplift of the Andes (Altiplano and Eastern Puna) (Fig. 3). The three environmental factors act in a correlated way on a large scale. The Cenozoic is characterised by a global trend of decrease of CO_2_ levels, which caused a decrease of the global temperature. This global cooling favoured the formation of polar ice caps, and consequently a decrease in global sea level^100,101^. However, there are patterns in the curve of the sea level that do not coincide with CO_2_ and temperature curves. This is because the changes in sea level in the short term are likely influenced by Milankovitch cycles, more than by CO_2_ levels^100^. On a regional scale, the climate was also influenced by the rise of the Andes. The Andes are the longest continental mountain belt on Earth and the highest mountain range outside of central Asia, with a complex geological history that changes along its latitude^20^. The Altiplano and the Eastern Puna are two of the eight geomorphological domains of the Central Andes (14–27°S)^21^, and it is between those latitudes that the highest peaks exist. Therefore, the uplift models of this area are crucial to understand which periods of the Andes orogenesis displayed high enough peaks to produce environmental changes. Paleoelevation reconstructions suggest a first pulse of elevation of the Central Andes between 30 and 20 Ma^22,23^. At this point (∼ 20 Ma), the Andes exceeded 50% of their current height and began to function as barriers of winds and moisture^102^. Then, between 10 and 6 Ma, the main phase of the Andean uplift occurred and present-day surface elevations over large areas were reached^23,103–106^. The Central Andes play an important role in influencing the climate by modifying the intertropical convergence zone, which defines the Pacific precipitation pattern, and influencing the moisture transport to South America^102,107^. The first aridification events of the Miocene are recorded since at least 15 Ma and increased between 6 and 3 Ma^108–110^. This implied the expansion of grasslands, steppes, and scrublands across South America and a concomitant restriction of forests, rainforests, and wetlands (including the Pebas Megawetland)^111–113^.

Our results indicate that all these changes (both globally and regionally) would have had an influence on the sparassodonts. Modern metatherians (*i.e.*, living marsupials) have a lactation period strongly synchronized with water and food availability, since it is the period when most of their energy is invested in their young and during which most of the development of the offspring occurs^114–116^. In addition, the body heat in marsupials derives from metabolic and muscular activity, whereas in placentals it is generated by brown adipose tissue^117^. Due to these physiological characteristics, the availability of food, which depends on temperature and rainfall^118^, plays a crucial role in marsupials. Ultimately, given the increased aridity caused by the rise of the Andes^39,119,120^ and the decrease in temperature^8,39^ due to low levels of CO_2_, sparassodonts would have become extinct due to their limitations in tolerance to environmental changes.

Here we highlight that a multilevel mixed model of evolution, where the evolutionary history of Sparassodonta is driven by a combination of intrinsic biotic factors (*i.e.*, body mass, diversity of Sparassodonta), extrinsic biotic factor (*i.e.*, diversity of Astrapotheria, Litopterna, other Metatheria, and Rodentia), and extrinsic abiotic factors (*i.e.*, CO_2_, sea level, temperature, and Andes uplift). Evolution operates in a pluralistic way, where all factors might prevail in different ways and at different times^29,121^, and as Jablonski^121^ remarks, trying to dichotomize macroevolutionary processes becomes counterproductive. Although we cannot claim if all these factors act independently or are interconnected as we expect, they were all crucial in the evolutionary history of sparassodonts.

## Material and methods

### Data set and palaeoenvironmental information

We compiled a data set of fossil occurrences for South American faunas including Sparassodonta, possible competing groups of predatory vertebrates (Carnivora, Phorusrhacidae, and carnivorous opossums), and other contemporaneous mammals (Artiodactyla, Astrapotheria, Litopterna, Microbiotheria, Notoungulata, Paucituberculata, Perissodactyla, Polydolopimorphia, Proboscidea, Pyrotheria, Rodentia, Xenarthra, and Xenungulata) (supplementary information 2). We used the database generated in previous works^7,8^ combined with the Paleobiology Database (paleobiodb.org, accessed until May 2021). Our data set focused on fossil occurrences found in South America and identified at the species level excluding all occurrences with uncertainty about taxonomy (i.e., those specimens marked with qualifiers such as cf., aff., and “’?”). Since the online database may contain errors, each entry was checked against the existing information in the literature and museum databases.

To test a putative competition with carnivorous opossums we built a variable with the species that traditionally are interpreted as ecologically comparable to sparassodonts: Sparassocynini, Thylatheridium, and Didelphini^7,8,39,42^. Since some groups had few occurrences, they were grouped together as following: foreign ungulates include Artiodactyla, Perissodactyla, and Proboscidea; other Metatheria include non-carnivorous opossums, Microbiotheria, Paucituberculata, and Polydolopimorphia; and South American native ungulates (SANUs) include Astrapotheria, Litopterna, Notoungulata, Pyrotheria, and Xenungulata. However, Astrapotheria, Litopterna, and Notoungulata were also evaluated separately because they have a relatively rich fossil record.

Particularly in the case of Sparassodonta, we generated four datasets, which allowed us to evaluate the impact of the fossil record on the analyses. First, the species occurrence dataset (*i.e.*, dataset of occurrences identified at the species level dated with precise methods). Occurrence is defined as the presence of a taxon at a certain stratigraphic level of a fossil locality. A given taxon will have several entries in the dataset if and only if it was recorded at different stratigraphic levels or different fossil locations. We define this dataset this way since in previous studies where this methodology is used, they incorporate the fossil record as occurrences^46,58,59^. Second, the fossil specimens dataset (*i.e.*, dataset of fossil specimens identified at the species level dated with precise methods). Every fossil specimen will be incorporated into the dataset no matter if it comes from the same stratigraphic level or from the same fossil location. We define this dataset this way since we consider it important to incorporate all the information that the fossil record gives us, including abundance. Third, the South American Age/Stage dataset (*i.e.*, dataset of occurrences identified at the species level dated with the boundaries of the South American Age/Stage). The entries are the same as the first dataset, but only the age ranges were modified for each occurrence. We use those age ranges since in previous studies of diversity curves, age is considered in this way^7,8,41^. Fourth, the genera dataset (*i.e.*, dataset of occurrences identified at the genus level dated with precise methods).

The final datasets of Sparassodonta are: species occurrence and South American Age/Stage datasets that include 66 species of Sparassodonta (157 occurrences), fossil specimens dataset that includes 352 fossil records for the 66 species, and genera dataset that includes 45 genera of Sparassodonta (163 occurrences). Additionally to these datasets, the rest of the fauna comprises: 36 species of Astrapotheria (97 occurrences), 85 species of Carnivora (401 occurrences), 34 species of carnivorous opossums (66 occurrences), 71 species of foreign ungulates (402 occurrences), 112 species of Litopterna (240 occurrences), 291 species of Notoungulata (766 occurrences), 164 species of other Metatheria (333 occurrences), 18 species of Phorusrhacidae (38 occurrences), 457 species of Rodentia (1154 occurrences), 477 species of SANUs (1155 occurrences), and 397 species of Xenarthra (1153 occurrences). It is noteworthy that fossil occurrences have a temporal uncertainty associated with the stratigraphic sequences where they were found. In some cases, the stratigraphic levels are dated using precise techniques (i.e., magnetostratigraphy, radioisotopes), while in other cases only by biostratigraphy. To reduce the bias, we included occurrences with a temporal range less than 15 Ma, and we constructed 20 databases of each clade where the ages of the occurrences were randomly resampled within each time range, following Silvestro et al.^45^.

We considered different environmental variables estimated for the Cenozoic of South America where possible; otherwise, global estimations were used. Our model includes: Altiplano, Eastern Cordillera, Eastern Puna, Western Cordillera, and Western Puna palaeoelevations^21^, global atmospheric CO_2_^101^, global atmospheric O_2_^122^, global δ^18^O as a proxy of temperature^123^, and global sea level^100^. The environmental variables were gathered from the raw values, and if not available, the values were extracted from the published graphs using GraphClick (Arizona Software, www.arizona-software.ch/graphclick).

### Speciation and extinction rates

The pattern of fossil occurrence is modelled by diversification processes (speciation and extinction) and preservation processes (fossilization and scientific sampling). Consequently, we estimated the parameters of the preservation process for each clade, the times of speciation (Ts) and extinction (Te) of each species, and the speciation and extinction rates of each clade and their variation through time, all within a hierarchical Bayesian framework using PyRate v3.0^124^. We approximated the posterior distributions of all parameters for each taxon through reversible jump Markov Chain Monte Carlo (RJMCMC)^57,125^, ran 20,000,000 MCMC generations, and sampled once every 5,000 to achieve convergence. We ran a maximum likelihood test to assess which preservation model is best supported by the data^57^, the model with the lowest AIC was non-homogeneous Poisson process of preservation (NHPP). Therefore, all analyses were set with a NHPP and accounted for varying preservation rates across taxa using the Gamma model. We monitored chain mixing and effective sample sizes by examining the log files in Tracer 1.7.1^126^ after excluding the first 20% of the samples as burn-in period. Posterior samples of the parameters from 20 randomized datasets were summarized by calculating their mean values and 95% credible intervals. We then used the estimated times of speciation and extinction of all species to carry out other analyses (see below).

The robustness of the PyRate approach has been thoroughly evaluated using simulations by Silvestro et al.^45,57^. Datasets were simulated under a range of potential biases, and the results showed that the dynamics of speciation and extinction rates are correctly estimated under a wide range of conditions, such as low levels of preservation, severely incomplete taxon sampling, and high proportion of singletons (see ref^45,57^ for more details of the simulations). In our case, the results showed that the mean preservation rates, averaged over 20 replicates, ranged across clades between 1.07 and 22.11 expected fossil occurrences per lineage per Ma, and the estimated heterogeneity parameters indicated considerable rate variation among the species of most clades (supplementary information 1, Table S3). Considering that our values range within the values used in previous works^45,46,59^, we can expect speciation and extinction rates to be reliably inferred by the birth–death analyses.

### Body mass correlated diversification

We tested if the diversification dynamics of Sparassodonta may be linked with changes in (log-transformed) body mass using a Covar birth–death model^124^. Body mass estimates of Sparassodonta were taken from the literature^8^. Values were obtained from postcranial, cranial, and dental measurements whenever possible (see ref^8^ for more detail).

We ran 20,000,000 MCMC iterations with a sampling frequency of 5,000 and combined the posterior samples of the parameters from the 20 replicates of both species occurrence dataset and fossil specimens dataset. However, since the Paleogene record is very limited, only species with Ts younger than 30 Ma were used. This model estimates correlation parameters (α_λ_, α_μ_) from the data, which were considered statistically significant when 0 was outside of the 95% credible intervals. Thus, α > 0 indicates a positive correlation between body mass and birth–death rates; α < 0 indicates a negative correlation.

### Multivariate correlations

We used a Multivariate Birth–Death model (MBD)^46^ to evaluate the correction of different external variables with speciation and extinction rates. We used Ts and Te of each species inferred from both the species occurrence dataset and the fossil specimens dataset. However, since the Paleogene record is very limited, only species with Ts younger than 30 Ma were used. We used as external variables the environmental factors mentioned above and the diversity trajectory calculated for the different taxa. All the trajectories were rescaled to vary between 0 and 1 before analyses (supplementary information 1, Fig. S2). We ran the MBD model using 200,000,000 MCMC generations and sampling every 50,000 to approximate the posterior distribution of all parameters. We summarized the results of the MBD analyses by calculating the posterior mean and 95% credible intervals of all correlation parameters (G_i_). The analysis also estimates the shrinkage weights (w) in order to distinguish if the correlation parameters are noise or signal (i.e., if w(G_i_) > 0.5, then G_i_ significantly differs from the background noise and represents a correlation)^46^. We ran both linear and exponential correlation models, and then we evaluated which model had more support by calculating log Bayes factors following Lehtonen et al.^46^. We mostly focus on the results obtained with the linear correlation models since Bayes factor indicated more support for the latter (BF = 2.21 for the species occurrence dataset, and BF = 13.60 for the fossil specimens dataset).

## Supplementary Information

Supplementary Information 1.

Supplementary Information 2.

## Data Availability

All data generated or analysed during this study are included in this published article (and its Supplementary Information files).
