# Supplementary material for: The multicausal twilight of South American native mammalian predators (Metatheria, Sparassodonta)
Source: Sci Rep. 2022 Jan 24;12:1224. doi: 10.1038/s41598-022-05266-z (PMC8786871; doi:10.1038/s41598-022-05266-z)
Supplement: Supplementary file 1 — Supplementary Information 1. [file 41598_2022_5266_MOESM1_ESM.docx]

Supplementary information 1 for:

THE MULTICAUSAL TWILIGHT OF SOUTH AMERICAN NATIVE MAMMALIAN PREDATORS (METATHERIA, SPARASSODONTA)

Fig. S1. The Bayesian estimates of speciation (blue), extinction (red), net diversification (grey, speciation minus extinction) rates, and diversity of South American Age/Stage dataset (a-c) and of genera dataset (d-f). Solid lines indicate mean rates, and the shaded areas show 95% credible intervals.


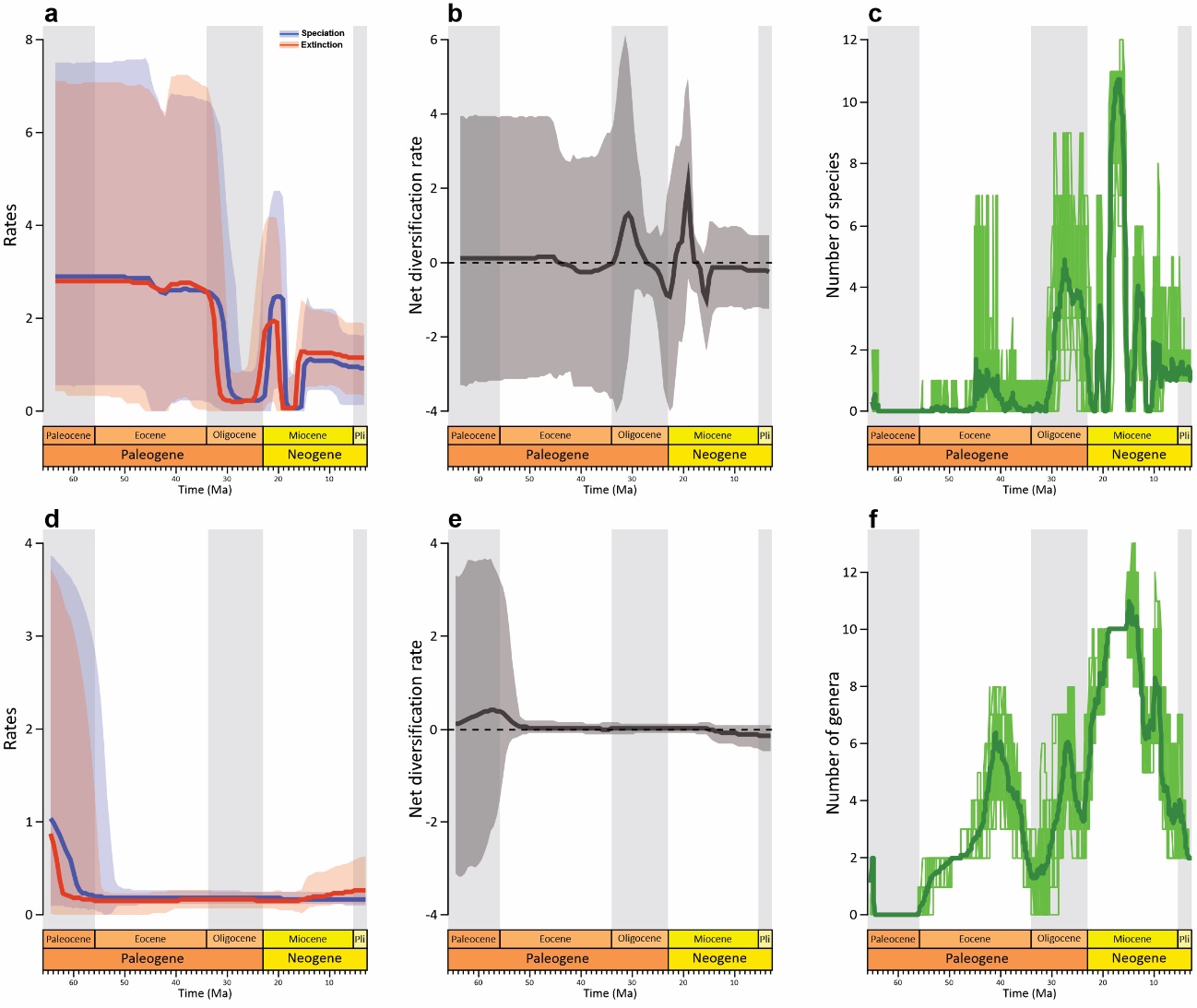


Fig. S2. Paleoenvironmental trajectories used in this study.


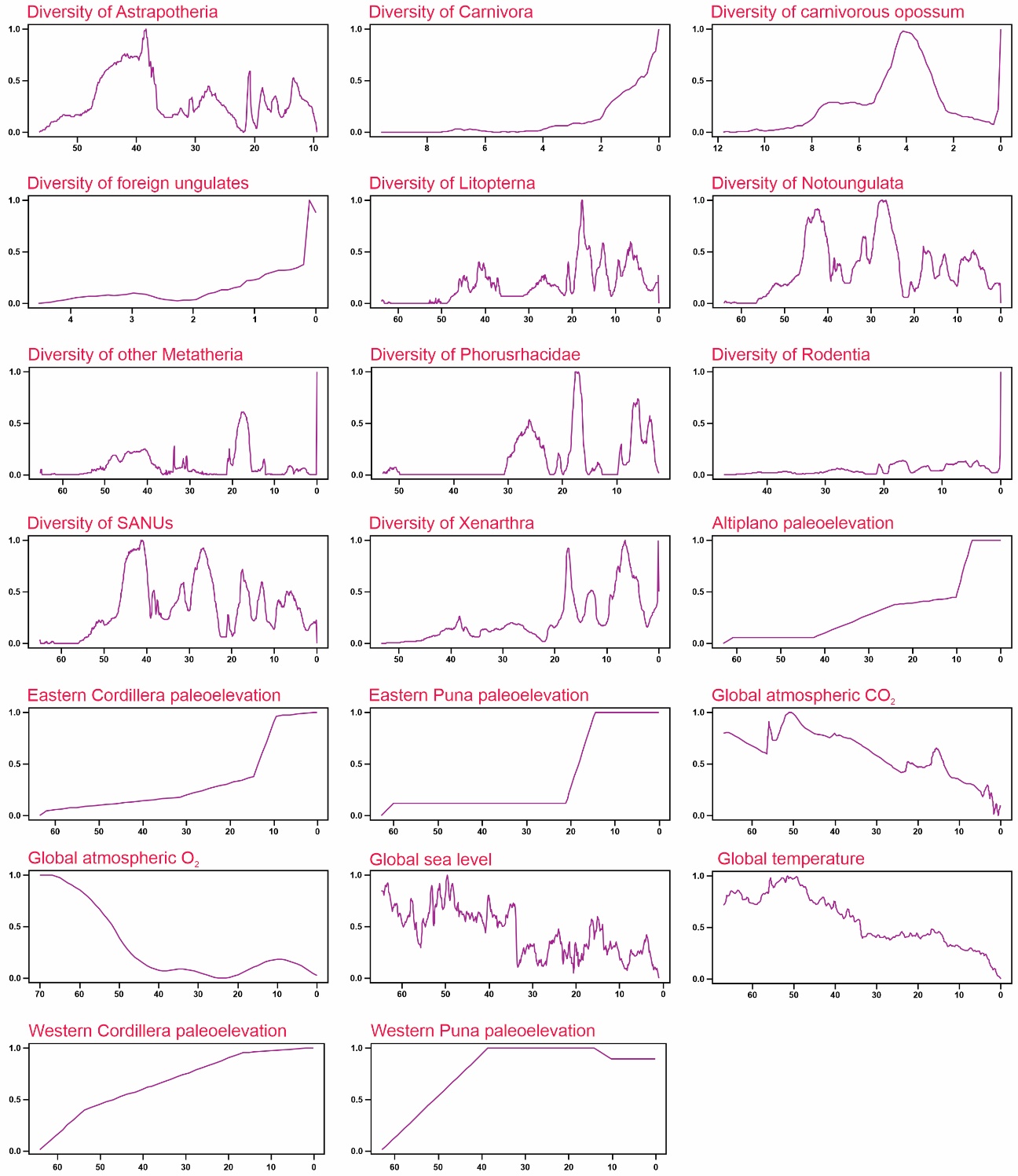


Table S1. Correlation parameters (G_i_) and shrinkage weights (W_i_) of the exponential and linear models with the species occurrence dataset. W_i_ greater than 0.5 (highlighted in bold) indicate significant evidence for correlation (shaded in grey).

|  | **Exponential model** | | | | **Linear model** | | | |
| --- | --- | --- | --- | --- | --- | --- | --- | --- |
|  | **Speciation rate** | | **Extinction rate** | | **Speciation rate** | | **Extinction rate** | |
|  | **G_l_** | **W_l_** | **G_m_** | **W_m_** | **G_l_** | **W_l_** | **G_m_** | **W_m_** |
| Diversity of Sparassodonta | -5.95 | **0.93** | 0.02 | 0.33 | -7.12 | **0.88** | 0.22 | 0.42 |
| Global atmospheric CO_2_ | 2.72 | **0.62** | 0.80 | 0.44 | 5.28 | **0.64** | 1.29 | 0.48 |
| Global atmospheric O_2_ | 0.57 | 0.41 | 0.20 | 0.40 | 0.93 | 0.47 | 0.67 | 0.47 |
| Global sea level | -1.79 | **0.53** | 0.08 | 0.39 | -2.87 | **0.57** | 0.45 | 0.49 |
| Global temperatura | 0.94 | 0.45 | 0.94 | 0.44 | 1.53 | **0.51** | 1.05 | 0.46 |
| Altiplano | -0.39 | 0.37 | 0.13 | 0.34 | -0.20 | 0.42 | 0.62 | 0.44 |
| Eastern Cordillera | -0.09 | 0.33 | -0.03 | 0.33 | -0.10 | 0.40 | 0.58 | 0.44 |
| Eastern Puna | -0.33 | 0.34 | 0.16 | 0.32 | -0.58 | 0.41 | 1.01 | 0.47 |
| Western Cordillera | 0.05 | 0.37 | 0.51 | 0.38 | 0.00 | 0.42 | 1.60 | 0.46 |
| Western Puna | 0.39 | 0.39 | 0.26 | 0.36 | 0.28 | 0.44 | 0.53 | 0.43 |
| Astrapotheria | 0.82 | 0.46 | -1.72 | **0.55** | 0.52 | 0.44 | -2.40 | **0.55** |
| Foreign ungulates | -0.32 | 0.42 | 0.09 | 0.41 | -0.67 | 0.47 | 0.33 | 0.46 |
| Litopterna | 3.72 | **0.70** | -1.13 | 0.48 | 4.60 | **0.65** | -3.45 | **0.61** |
| Notoungulata | -0.21 | 0.36 | -0.50 | 0.39 | -0.42 | 0.43 | -0.39 | 0.42 |
| Other Metatheria | 0.92 | 0.44 | -1.91 | **0.59** | 0.35 | 0.44 | -3.05 | **0.59** |
| Rodentia | 4.03 | **0.52** | 0.45 | 0.41 | 8.01 | **0.54** | -0.16 | 0.47 |
| SANUs | -0.56 | 0.39 | -0.67 | 0.42 | -0.60 | 0.43 | -0.97 | 0.45 |
| Xenarthra | -0.66 | 0.43 | 0.31 | 0.36 | -1.08 | 0.47 | 0.07 | 0.41 |
| Carnivorous opossum | 0.15 | 0.34 | -0.24 | 0.33 | 0.52 | 0.40 | -0.32 | 0.41 |
| Carnivora | -0.85 | 0.42 | -0.02 | 0.38 | -1.65 | 0.49 | 0.35 | 0.45 |
| Phorusrhacidae | -0.47 | 0.40 | -0.84 | 0.45 | -0.60 | 0.42 | -1.19 | 0.47 |

Table S2. Correlation parameters (G_i_) and shrinkage weights (W_i_) of the exponential and linear models with the fossil specimens dataset. W_i_ greater than 0.5 (highlighted in bold) indicate significant evidence for correlation (shaded in grey).

|  | **Exponential model** | | | | **Linear model** | | | |
| --- | --- | --- | --- | --- | --- | --- | --- | --- |
|  | **Speciation rate** | | **Extinction rate** | | **Speciation rate** | | **Extinction rate** | |
|  | **G_l_** | **W_l_** | **G_m_** | **W_m_** | **G_l_** | **W_l_** | **G_m_** | **W_m_** |
| Diversity of Sparassodonta | -6.73 | **0.96** | 0.74 | 0.41 | -6.30 | **0.90** | 1.45 | 0.49 |
| Global atmospheric CO_2_ | 2.63 | **0.64** | 0.48 | 0.41 | 1.98 | **0.52** | 0.55 | 0.40 |
| Global atmospheric O_2_ | 0.91 | 0.43 | 0.01 | 0.41 | 2.17 | 0.49 | -0.26 | 0.45 |
| Global sea level | -1.06 | 0.47 | 1.21 | **0.51** | -1.19 | 0.46 | 0.68 | 0.43 |
| Global temperatura | 0.74 | 0.45 | 0.67 | 0.43 | 0.99 | 0.45 | 0.35 | 0.41 |
| Altiplano | -0.64 | 0.41 | 0.00 | 0.34 | -1.61 | **0.50** | 0.07 | 0.38 |
| Eastern Cordillera | 0.30 | 0.36 | -2.32 | 0.35 | 0.43 | 0.38 | 0.02 | 0.38 |
| Eastern Puna | -0.49 | 0.36 | 0.72 | 0.43 | -0.09 | 0.35 | 2.04 | **0.57** |
| Western Cordillera | 0.12 | 0.38 | 0.50 | 0.39 | 0.46 | 0.40 | 0.93 | 0.41 |
| Western Puna | 0.38 | 0.40 | 0.07 | 0.36 | 0.50 | 0.39 | 0.06 | 0.38 |
| Astrapotheria | 1.34 | **0.54** | -1.43 | **0.54** | 1.79 | **0.52** | -1.10 | 0.47 |
| Foreign ungulates | -0.12 | 0.43 | 0.54 | 0.44 | 0.08 | 0.44 | 1.03 | 0.45 |
| Litopterna | 3.61 | **0.77** | -1.22 | 0.48 | 1.89 | **0.55** | -2.48 | **0.55** |
| Notoungulata | -0.09 | 0.34 | -1.14 | **0.50** | -0.05 | 0.34 | -0.87 | 0.43 |
| Other Metatheria | 0.73 | 0.44 | -1.54 | **0.52** | 1.35 | **0.50** | -2.08 | **0.54** |
| Rodentia | 1.08 | 0.45 | -0.28 | 0.43 | 5.98 | **0.54** | -1.58 | 0.47 |
| SANUs | -0.42 | 0.38 | -0.94 | 0.48 | -0.20 | 0.37 | -0.63 | 0.42 |
| Xenarthra | -0.45 | 0.39 | 0.10 | 0.36 | -0.37 | 0.38 | -0.39 | 0.40 |
| Carnivorous opossum | -0.09 | 0.35 | 0.07 | 0.33 | 0.03 | 0.35 | 0.11 | 0.36 |
| Carnivora | -0.67 | 0.43 | 0.19 | 0.41 | -0.71 | 0.44 | 0.72 | 0.43 |
| Phorusrhacidae | -0.10 | 0.35 | -1.14 | **0.51** | 0.29 | 0.37 | -0.88 | 0.44 |

Table S3. Posterior estimates of the preservation rate (expected number of occurrences per lineage per *Ma*) and heterogeneity parameter (shape parameter of the Gamma distribution). Lower and upper bounds of the 95% credible intervals are provided.

| Clade | Preservation rate (q) | Heterogeneity parameter (α) |
| --- | --- | --- |
| Sparassodonta (species occurrence dataset) | 1.21 (0.80 – 1.67) | 2.88 (0.42 – 7.74) |
| Sparassodonta (South American Age/Stage dataset) | 1.51 (0.99 – 2.09) | 2.23 (0.45 – 5.22) |
| Sparassodonta (fossil specimens dataset) | 4.17 (3.24 – 5.11) | 0.84 (0.51 – 1.13) |
| Sparassodonta (genera dataset) | 0.65 (0.45 – 0.86) | 2.00 (0.61 – 3.80) |
| Astrapotheria | 1.07 (0.66 – 1.51) | 0.81 (0.22 – 1.50) |
| Carnivora | 7.84 (5.79 – 10.05) | 1.03 (0.46 – 1.52) |
| Carnivorous opossum | 1.17 (0.63 – 1.69) | 10.51 (2.33 – 19.98) |
| Foreign ungulates | 22.11 (17.56 – 26.79) | 0.55 (0.29 – 0.87) |
| Litopterna | 1.18 (0.87 – 1.50) | 1.16 (0.58 – 1.82) |
| Notoungulata | 1.14 (0.95 – 1.33) | 0.69 (0.38 – 1.08) |
| Other Metatheria | 1.55 (1.18 – 1.93) | 0.93 (0.49 – 1.46) |
| Phorusrhacidae | 1.14 (0.53 – 1.86) | 9.46 (0.87 – 19.14) |
| Rodentia | 4.02 (2.73 – 5.33) | 0.68 (0.33 – 0.91) |
| SANUs | 1.21 (1.04 – 1.39) | 0.63 (0.40 – 0.89) |
| Xenarthra | 5.54 (4.32 – 6.79) | 0.33 (0.27 – 0.40) |
